# Affordability Analysis of Selected Medicines in Iran: A National Cross-sectional Survey Using World Health Organization Out-of-Pocket Methodology

**DOI:** 10.5812/ijpr-163774

**Published:** 2026-02-15

**Authors:** Fatemeh Soleymani, Amirhossein Abdi, Alireza Mirzaei, Mohammad Mahdi Raeis Zadeh, Ali Zeinalkhani, Behzad Fatemi

**Affiliations:** 1 Department of Pharmacoeconomics and Pharma Management, School of Pharmacy, Tehran University of Medical Sciences, Tehran, Iran; 2 Pharmaceutical Management and Economics Research Center (PMERC), Institute of Pharmaceutical Sciences (TIPS), Tehran University of Medical Sciences, Tehran, Iran

**Keywords:** Out-of-Pocket Payments, Medication Costs, Catastrophic Health Expenditure, Impoverishing Health Expenditure, Health Insurance Coverage

## Abstract

**Background:**

Increased costs of prescription drugs impose tremendous economic burdens on patients globally, which usually result in low drug adherence and poor health outcomes. In Iran, out-of-pocket (OOP) expenditures are severe, particularly among vulnerable groups such as the elderly. This analysis reviews OOP expenditures of 51 medications in Iran to provide useful insights regarding healthcare policy.

**Objectives:**

To analyze OOP payments for medications in Iran during a seven-year period, revealing trends, financial needs, and policy requirements that increase financial protection and drug access.

**Methods:**

We included 51 medicines, meeting the WHO/HAI minimum sample size requirement for reliability (n = 50), consisting of the universal core list (n = 14) and a supplementary list based on local disease prevalence (n = 37). Following RECORD guidelines for observational studies, data were sourced from the two largest national health insurance funds [Social Security Organization (SSO), Iran Health Insurance Organization (IHIO)], covering the majority of the Iranian population, and pharmacy records from 2016 to 2022. OOP costs, insurance coverage, and affordability were analyzed using impoverishing health expenditure (IHE) and catastrophic health expenditure (CHE) metrics. To control for economic confounders, costs were adjusted using purchasing power parity (PPP). Joinpoint regression assessed annual OOP changes, with significance set at P-value < 0.05.

**Results:**

Over the seven-year period, PPP-OOP payments showed a non-significant decreasing trend [average annual percent change (AAPC): -1.2% for SSO; -1.38% for IHIO]. Despite this, OOP expenditure remained high, averaging approximately 40% of total medication costs. Significant heterogeneity was observed; for instance, Spironolactone saw the highest cost increase (AAPC +8.29%), while Insulin Glargine showed the largest decrease (AAPC -12.59%). High OOP payments were observed for medications like dexamethasone and chlorpromazine, while insulin and clozapine also carried high costs under insurance. The variability in OOP expenses across medicines highlights differences in pricing and insurance policies. Catastrophic expenses were prominent for certain medications, such as enoxaparin, especially at higher expenditure thresholds based on the 40% capacity-to-pay metric. Limitations included the use of administrative claims data subject to potential operational errors and the restriction of the sample to 51 medicines, which may limit generalizability to the broader pharmaceutical market.

**Conclusions:**

This research reveals high OOP spending for Iranian patients, with considerable drug-specific heterogeneity. The implications suggest that policy action in terms of price reform and wider insurance coverage is needed to minimize financial burden and ensure access to medicines. Further studies are required to determine the impact of high OOP spending on patient adherence and to inform specific health policies in Iran and beyond.

## 1. Background

Regardless of cultural, economic, or healthcare differences, the rising cost of prescription medicines is a global concern (1). Healthcare expenses, particularly for medicines, place a heavy burden on populations worldwide, irrespective of the healthcare scheme or insurance coverage. Many systems use “cap” and co-payment cost-sharing to curb spending: A cap sets the insurer’s maximum payment over a period, and a co-payment is a fixed amount the patient pays per service or prescription. Such policies influence medicine use, healthcare utilization, health outcomes, and total spending (2). Rising healthcare costs are increasingly recognized as a major barrier to patients’ ability to pay, particularly in developing nations. In Iran, for example, out-of-pocket (OOP) payments represent over 50% of healthcare expenditure, and informal patient charges remain prevalent, further exacerbating financial hardship (3). This financial strain is not limited to general care but is also evident in the management of specific major diseases, such as breast cancer, where patients continue to bear a substantial portion of treatment costs directly (4). Recent studies have shown that high OOP medication costs increase financial pressure and are linked to poor drug adherence and worsening health outcomes (5, 6). In studies, it is found that high OOP costs result in poor drug adherence and worsening health conditions (2). Iranian patients continue to face heavy financial strain from high OOP payments for medicines (3). Older patients face a very high economic burden for their need for drugs, as a large share of prescription charges is paid OOP (6). Older adults bear a particularly high economic burden because a large share of prescription costs is paid OOP. A 2019 study from Shiraz (7) shows this framework disproportionately harms lower-income groups, indicating a regressive pattern (7). Out-of-pocket payments for medication are a major driver of patient impoverishment globally, though the extent varies by country; the World Health Organization (WHO) has highlighted this problem (8). In Europe and the United States, higher OOP spending correlates with lower medication adherence and slower uptake of new treatments, particularly among older and low-income patients; shifts toward off-patent drugs may reduce total spending but also reduce dispensed prescriptions (9). Across South Asia, OOP payments for medicines are a major source of financial hardship; in India, total OOP health spending comprises approximately 70% of total health expenditure and can push households below the poverty line, particularly following hospitalization and when branded medicines are chosen over generics (10). In Nepal and Bangladesh, catastrophic OOP payments for medication disproportionately affect the poor, elderly, and chronically ill (11, 12). The World Health Organization therefore emphasizes policies that strengthen financial protection and improve affordability (13).

In Iran, OOP payments remain a persistent challenge despite efforts to expand insurance coverage and subsidize care. High OOP spending disproportionately affects vulnerable groups — including the elderly, low-income households, and patients with chronic conditions (6, 14). Iran’s Health Transformation Plan (HTP, launched in 2014) sought to reduce OOP payments and catastrophic health expenditures (CHE) by expanding coverage and capping hospital payments (15). However, pharmaceutical costs remain a major source of financial hardship, exacerbated by high inflation, sanctions-related shortages, and reliance on imported medicines (16).

## 2. Objective

Accordingly, this study estimates OOP payments for 51 medicines in Iran and assesses alignment with World Health Organization recommendations for financial protection. Situating Iran within international debates on affordability, we propose practical measures to strengthen cost-sharing design — such as equitable drug-pricing policies — and to enhance protection for vulnerable groups.

## 3. Methods

### 3.1. Study Design

This retrospective cross-sectional study uses an observational study design, utilizing collected data to evaluate the OOP payments for medicines in Iran. The method used in our study follows the Strengthening the Reporting of Observational Studies in Epidemiology (STROBE) guidelines for cross-sectional studies, as extended by the REporting of studies Conducted using Observational Routinely-collected Data (RECORD) statement. This ensures comprehensive reporting of the observational design while addressing specific requirements for routinely collected health data.

We calculated the insurances' and patients' share of payments for each medicine in the follow-up period (2016 - 2022). Iran’s health insurance landscape comprises four major schemes: (1) The Social Security Organization (SSO/SSIO), ~27 million beneficiaries; (2) the Iran Health Insurance Organization (IHIO), ~39 million; (3) the Armed Forces Medical Services Insurance Organization (AFMSIO), > 4 million; and (4) the Imam Khomeini Relief Committee Health Insurance (ICHI), ~4.5 million disadvantaged individuals (3). Our analysis focused on the two largest insurers (SSO and IHIO), which together cover the majority of the population; AFMSIO and ICHI were not included due to data unavailability.

For each medicine and each year, we obtained the official market tariff (private sector price without insurance coverage) set annually by the Ministry of Health and the Food and Drug Administration (IFDA), as well as the insurer-specific reimbursement tariffs for SSO and IHIO. Based on these values, we calculated the final insured price under each insurer. The patient’s OOP payment was determined as the sum of the insurer co-payment (franchise) on the official tariff, the difference between the market and insurer tariffs, and the approved injection administration fee (for injectable medicines). OOP payment was expressed as a percentage of the total medicine utilization cost in Iran’s public and private health sectors (65/35%, respectively) (17).

#### 3.1.1. Variables and Measurement

The primary outcome variable was the OOP payment, defined as the direct payment made by the patient at the point of service. For each medicine, OOP was calculated using the following formula: OOP = (P_market_ – P_reimbursed_) + Franchise + C_admin_, Where: P_market_ is the official market tariff (private sector price); P_reimbursed_ is the insurer-specific reimbursement tariff; Franchise is the patient's statutory co-payment rate (10 - 30%); and C_admin _is the approved technical and administration fee (e.g., injection fees).

The OOP expenditures were calculated in Iranian Rials and then converted to U.S. dollars using a Purchasing Power Parity (PPP) Index that we calculated separately for each year from 2016 to 2022. This approach ensured that the conversion reflected annual changes in relative price levels rather than applying a single average rate across the entire study period.

### 3.2. Setting and Data Scope

The study setting encompassed the entire Iranian pharmaceutical market, aggregating data from both public (65% of market share) and private (35%) dispensing sectors. Data were extracted covering a seven-year period from March 20, 2016, to March 20, 2022 (corresponding to the Iranian calendar years 1395 - 1400). The analysis focused on the insured population covered by the SSO and IHIO, which together insure the majority of the Iranian population.

### 3.3. Sample Size and Selection of Medicines

The study utilized data from sources like national health insurance databases (which provide information on pharmaceutical coverage and reimbursement rates), retail pharmacy records (to obtain information on average pharmaceuticals market prices), and the Iranian National Statistical Organization (INSO) to gather socioeconomic information about Iran’s population. The study sample size was determined a priori based on World Health Organization/Health Action International (WHO/HAI) guidelines, which recommend assessing a minimum of 50 medicines to ensure a representative and feasible cross-sectional analysis of the pharmaceutical market.

### 3.4. Data Collection

For each of the 51 selected medicines, three main categories of data were collected for each study year (2016 - 2022): The mean retail market price (private sector price without insurance coverage) obtained from pharmacy sales records; the insurance co-payment rate (franchise) and the mean reimbursed price under both major insurers extracted from official insurance databases; and the administration cost, including injection fees, consumables, and pharmacist service charges, as set in the national service tariff schedule. All tariffs and reimbursement rates were based on government-regulated pricing, which is applied uniformly across the country. Moreover, to assess patients' affordability, socioeconomic data of the Iranian population were collected from INSO. All data collection processes were performed using a predesigned Excel form based on Health Action International (HAI) guidelines for price, availability, and affordability of medicines. The aim of this study was limited to evaluating OOP payments for patients covered by these two main insurers and did not include other sources of medicine procurement such as charity organizations or similar providers. It should be noted that no systematic data were available for actual costs in charity pharmacies or in private facilities operating outside the standardized tariff framework; therefore, these could not be incorporated into the model. To minimize selection bias, we utilized dual-selection criteria for medicines (World Health Organization core list and local prevalence) and cross-verified pricing data between national insurance databases and private pharmacy records to reduce information bias related to tariff discrepancies.

### 3.5. Eligibility Criteria

#### 3.5.1. Inclusion Criteria

Inclusion criteria for participants encompassed all insured individuals covered by the SSO and IHIO who received prescriptions for the selected medicines between 2016 and 2022. According to the World Health Organization’s handbook Measuring Medicine Prices, Availability, Affordability and Price Components (2nd Edition), medicine price surveys should assess up to 50 medicines to ensure feasibility. This was done to standardize and allow for the comparison of studies on medicine availability, prices, and affordability. The medicines are chosen from three main sources: (1) The universal core list, consisting of 14 World Health Organization/Health Action International essential core medicines that are required in all surveys to allow cross-national comparisons; (2) a regional core list of 16 medicines, customized to regional health priorities while enabling comparisons within the region; and (3) a country-specific additional list of at least 20 medicines, selected according to local disease prevalence and healthcare system requirements (18). The World Health Organization suggests looking into a minimum of 50 medicines for these studies. The list of 14 core medicines is available in Appendix 1 in Supplementary File 1.

In the present study, in addition to the 14 core medicines, 37 other medicines were selected, for a total of 51 study medicines (14 World Health Organization/Health Action International core; 37 supplementary). These selections were based on the World Health Organization's essential medicines list and customized to fit the Iranian situation, taking into account disease patterns and the importance of treatments. A complete list of 14 core medicines and 37 selected medicines is provided in Appendix 2 in Supplementary File 1.

#### 3.5.2. Exclusion Criteria

To ensure data consistency and reliability, the following exclusions were applied: Excluded Insurance Schemes: Beneficiaries covered by the Armed Forces Medical Services Insurance Organization (AFMSIO) and the Imam Khomeini Relief Committee (ICHI) were excluded due to the unavailability of accessible longitudinal data. Excluded Settings: Prescriptions dispensed in charity pharmacies or private facilities operating outside the standardized national tariff framework were excluded, as systematic pricing data for these non-standardized sectors were unavailable. Records with missing or unverifiable data points (e.g., undefined dispensing dates or incomplete cost fields) were removed from the final analysis to prevent calculation errors.

### 3.6. Calculation of the Affordability of Medicines

In assessing the affordability of medicines, we focused on two key parameters: Impoverishing health expenditure (IHE) and CHE. If a patient's OOP expenditure for a cycle of acute diseases or a month of chronic diseases exceeded their daily income, it was categorized as an IHE (19). Additionally, we employed a descriptive-analytical method aligned with the World Health Organization's standards to calculate the incidence of CHE, using a threshold of 40% of the household's capacity to pay (after subtracting subsistence expenditures such as food, housing, and utilities), as recommended by the World Health Organization for assessing financial protection in vulnerable populations (15). Other thresholds (10% and 25%) were not applied, as the analysis focused solely on the 40% capacity to pay metric to reflect the financial burden post-basic needs. Our evaluation encompassed OOP payments, IHE expenditures, and CHE, stratified by insurance organization coverage and urban/rural subpopulations.

### 3.7. Analytical Approach

This study employed the Joinpoint regression method to assess the annual percentage change (APC) in OOP expenditure for pharmaceutical utilization in Iran from 2016 to 2022. All analyses were conducted using Microsoft Excel and Joinpoint regression version 5.0.2. Statistical significance was determined with a threshold of P-value < 0.05. To control for the confounding effect of temporal inflation and currency devaluation during the seven-year observation period, all nominal costs were adjusted using annual PPP conversion factors.

## 4. Results

**Figure 1.**
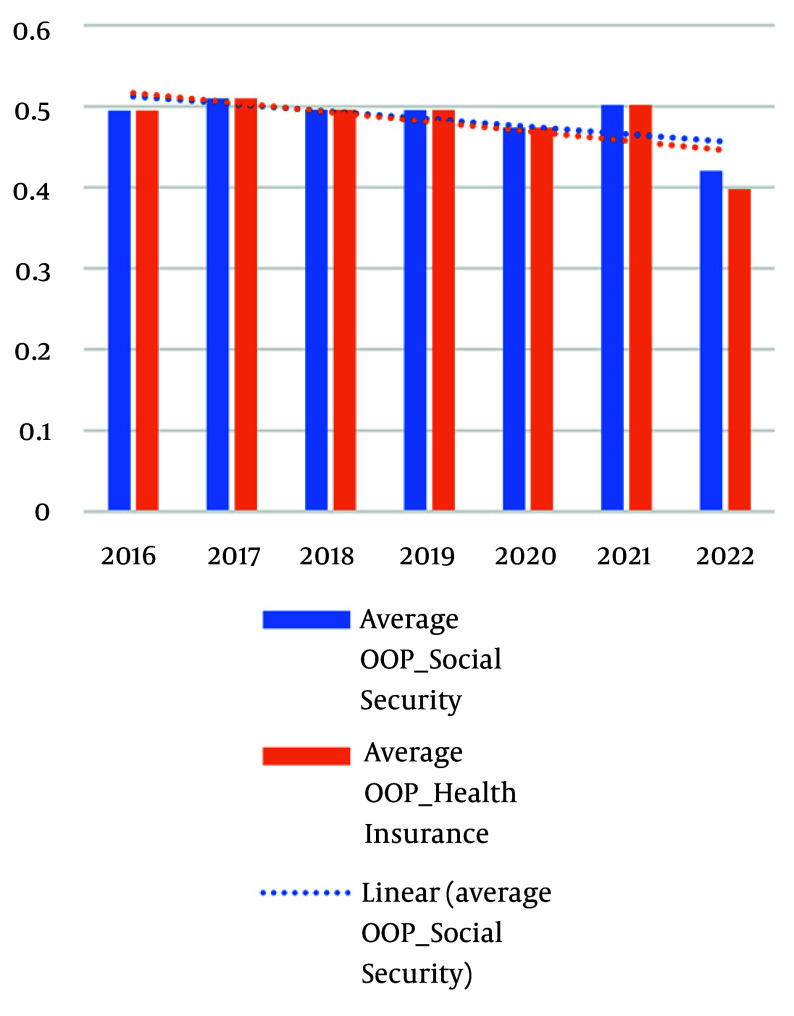
Average purchase of drugs with out-of-pocket (OOP) payments compared to purchases of medicines with Social Security and Health Insurance Organizations 2016 - 2022 National Minimum Wage (USD) 22.95$, 23.73$, 21.94$, 21.47$, 19.32$, 17.53$, 20.60$. Enoxaparin pen cost/ 1 cycle of treatment (USD) 72.65, 75.36, 64.16, 69.13, 53.94, 67.09, 60.54. Insulin glargine pen cost/ 1 cycle of treatment (USD) 36.86, 34.37, 27.53, 21.87, 15.46, 13.95, 11.53.

In the seven-year follow-up of this study, considering purchasing and administration costs, the OOP payments showed a decreasing trend. However, this decrease was not statistically significant, and it remained around 40% under both major insurance organizations in Iran, aligning with the 40% capacity to pay threshold used throughout the analysis (Figure 1). 

According to the National Minimum Wage (NMW) for daily employees in Iran from March 2016 to March 2022, patients' OOP payments for enoxaparin 4000 IU/0.4mL in all the studied years and insulin glargine during the years from 2016 to 2019, faced patients with the IHE health expenditures (Table 1). 

**Table 1.** National Minimum Wage vs. Out-of-Pocket Costs for Enoxaparin and Insulin Glargine (2016 - 2022), Highlighting Impoverishing Health Expenditure Risk ^a^

Variables	2016	2017	2018	2019	2020	2021	2022
**National minimum wage**	22.95	23.73	21.94	21.47	19.32	17.53	20.60
**Enoxaparin pen cost/1 cycle of treatment**	72.65	75.36	64.16	69.13	53.94	67.09	60.54
**Insulin glargine pen cost/1 cycle of treatment**	36.86	34.37	27.53	21.87	15.46	13.95	11.53

^a^ Costs are in USD (PPP-adjusted).

Considering average seven-year purchasing and administration costs, as well as insurance coverage for all studied medications, the highest percentage of OOP payments were associated with spironolactone tablets, dexamethasone ampoules, and prednisolone tablets with 92.3%, 86.5%, and 85.67%, respectively, under SSO coverage. Interestingly, the highest percentage of OOP under IHIO coverage were carvedilol tablets, clozapine tablets, and insulin regular pen with 91.80%, 86.50%, and 85.60%, respectively. Conversely, the lowest percentage of OOP payments was observed for metformin tablets, lithium carbonate tablets, and sodium valproate tablets with 19.8%, 22.90%, and 23.70% under the SSO coverage. In addition, under IHIO coverage, the lowest percentage of OOPs were related to sulfasalazine tablets, digoxin tablets, and fluoxetine capsules with 12.90%, 13.9%, and 19.8%. The Supplementary File 2 details OOP cost percentages for included medications from 2016 to 2022 (Supplementary File 2).

The OOP expenditures for all 51 studied medicines in Iran were calculated using the PPP index, with complete results provided in Appendix 3 in Supplementary File 1. During these years, the highest average monthly purchasing costs among the studied medicines were associated with enoxaparin ($152.1), insulin glargine ($61), and lithium carbonate ($35.6). Conversely, the lowest average monthly costs were observed for diazepam ($0.41), diclofenac ($1.1), and cetirizine ($1.23). It is important to note that these costs exclude administration and other direct or indirect expenditures.

According to the Joinpoint regression analysis, the average annual percent change (AAPC) for OOP expenditures under SSO was -1.2 (95% CI: -3.26 to 0.70), while under IHIO coverage, it was -1.38 (95% CI: -3.6 to 0.71). Nevertheless, these differences did not achieve statistical significance (Table 2). 

**Table 2.** Annual and Average Annual Percent Change (APC/AAPC) in Out-of-Pocket Payments Social Security Organization and Iran Health Insurance Organization (2016 - 2022) ^a^

Cohort (Joinpoints)	Metric	Range	Lower - Upper Endpoint	Estimate (APC/AAPC)	Lower-Upper CI	Significance
**SSO - 0**	APC	Segment 1	2016 - 2022	-1.1953	-3.2579, 0.7044	Not significant
**SSO - 0**	AAPC	Full Range	2016 - 2022	-1.1953	-3.2579, 0.7044	Not significant
**IHIO - 0**	APC	Segment 1	2016 - 2022	-1.3752	-3.6492, 0.7141	Not significant
**IHIO - 0**	AAPC	Full Range	2016 - 2022	-1.3752	-3.6492, 0.7141	Not significant

Abbreviations: APC, annual percent change; AAPC, average annual percent Change; CI, confidence interval.

^a^ Neither APC nor AAPC values were statistically significant (alpha = 0.05). Test statistics and P-values were unavailable for the empirical quantile method.

Considering a threshold of 10% of household payment capacity, the use of insulin (glargine pen) and enoxaparin 4000 IU/0.4mL, between 2016 and 2022, faced patients with CHE. When evaluating based on the thresholds of 25% and 40% household payment capacity, both insulin glargine and enoxaparin caused CHE throughout the entire study period.

Among all studied medicines, the AAPC of OOP payments for ezetimibe and chlorpromazine ampoule were increasing and statistically significant during the years 2016 to 2022 under SSO and IHIO. At the same time, the AAPC of OOP payment for carbamazepine, enoxaparin, clopidogrel, lithium, fluoxetine, biperiden, sulfasalazine, beclomethasone spray, ibuprofen, metformin, propranolol, isosorbide dinitrate, sodium valproate, glargine, and regular insulins were decreasing and statistically significant (Appendix 4 in Supplementary File 1).

Among studied medicines, spironolactone showed the highest AAPC of 8.29 (95% CI: -0.69 to 15.79). In comparison, insulin glargine pen had the lowest AAPC of -12.59 (95% CI: -19.36 to -3.28), which indicates that the greatest increase in OOP was related to spironolactone medicine and the greatest decrease in OOP was related to insulin glargine pen (Appendix 4 in Supplementary File 1).

## 5. Discussion

This cross-sectional study shows substantial differences in mean OOP drug expenditures in Iran from 2016 to 2022, indicating economic burden and potential access problems. Excessive OOP costs — especially for long-term treatment of chronic diseases — create barriers to starting and continuing care, undermining adherence and outcomes and underscoring the need for timely policy action to reduce patient costs, particularly in resource-constrained settings (20).

High prices for some medicines raise concerns about affordability and accessibility in Iran, whereas older generics (e.g., diazepam, diclofenac, cetirizine) are relatively affordable. This discrepancy supports targeted cost-containment to make newer medicines affordable and improve adherence and outcomes. Broader affordability is essential for equitable access (2).

Notwithstanding some reduction in insurance, Iranian OOP spending is high at the estimated 40%, which attests to the continued financial burden for patients. This is in keeping with other earlier work, e.g., Heidari et al., where attempts to limit OOP cost have not succeeded on account of insufficient insurance cover and increased healthcare spending. The continued financial burden highlights the importance of more advanced policy intervention to reduce the cost burden for patients, especially for noncommunicable diseases (21).

Our seven-year analysis highlights substantial variation in OOP payments across drugs, reflecting both price differences and the complexity of insurance coverage. The higher OOP share for cheaper drugs suggests that major insurers prioritize coverage for high-cost products. While this may improve access to expensive therapies, it raises concerns about the adequacy of coverage for lower-cost yet essential medicines.

Specific medicines — notably ezetimibe and chlorpromazine ampoules — showed rising OOP spending from 2016 to 2022. Such increases threaten adherence and outcomes and point to underlying pricing and reimbursement issues that warrant policy action to restore affordability and protect health.

Trends in OOP spending varied widely across drug classes. Although OOP spending declined within SSO and IHIO coverage, the changes were not statistically significant. Patterns also differed by insurance status, socioeconomic position, and clinical need among disadvantaged groups (e.g., older adults). These findings underscore the multifactorial nature of OOP spending and the need for policies that address these determinants to reduce financial strain on vulnerable communities.

### 5.1. Limitations

A number of limitations must be considered when interpreting our results. Firstly, the research made use of routinely available electronic information, which might contain operational errors. These errors have the potential to affect the completeness and accuracy of our data and, therefore, our analysis results. In an effort to respond to such challenges, we made use of the latest available data and verified our results using alternative data sources where possible. Second, according to World Health Organization standards, few drugs were used in this study. The selection does not account for all drugs being sold in Iran, and therefore there might be a limitation to the generalizability of our results to the entire Iranian pharmaceutical market.

### 5.2. Conclusions

The study highlights economic challenges and healthcare access barriers faced by Iranian patients, demonstrating significant variations in OOP medication expenditures. While there is a modest decline in aggregate OOP spending, significant variation exists among particular medicines, pointing to the necessity of context-specific policy interventions. The results necessitate continued monitoring of drug prices and insurance coverage to create strategies that minimize economic burdens and provide equitable access to necessary medications. Future studies need to investigate the effects of high OOP payments on patient compliance and health outcomes and investigate alternative approaches to construct a more financially stable healthcare system in Iran.

ijpr-25-1-163774-s001.zip

## Data Availability

The dataset presented in the study is available on request from the corresponding author during submission or after publication.
