# Supplementary material for: Affordability Analysis of Selected Medicines in Iran: A National Cross-sectional Survey Using World Health Organization Out-of-Pocket Methodology
Source: Iran J Pharm Res. 2026 Feb 15;25(1):e163774. doi: 10.5812/ijpr-163774 (PMC13181218; doi:10.5812/ijpr-163774)
Supplement: ijpr-25-1-163774-s001.zip [file ijpr-25-1-163774-s001.zip › -ijpr-25-01-163774-s001.pdf]

Appendix 1 presents the mandatory global core list of medicines required by World Health Organization (WHO) methodology for cross-country comparability in affordability studies. It includes therapeutic categories, generic names, strengths, dosage forms, and primary indications for each medicine, serving as the standardized foundation for international benchmarking.

**Number**	**Drug category**	**Generic name**	**Strength**	**Dosage form**	**Disease**
**1**	Antidepressant (Tricyclic)	Amitriptyline	25 mg	Tablet	Neurology
**2**	Antibiotic (Penicillin)	Amoxicillin	500 mg	Capsule	Antibiotics
**3**	Cardiovascular Drug (Beta-Blocker)	Bisoprolol	5 mg	Tablet	Cardiovascular
**4**	Antihypertensive (ACE Inhibitor)	Captopril	25 mg	Tablet	Cardiovascular
**5**	Antibiotic (Cephalosporin)	Ceftriaxone	1 g	Vial	Antibiotics
**6**	Antibiotic (Fluoroquinolone)	Ciprofloxacin	500 mg	Tablet	Antibiotics
**7**	Antibiotic (Sulfonamide combination)	Cotrimoxazole	8+40 mg/ml	Suspension	Antibiotics
**8**	Benzodiazepine	Diazepam	5 mg	Tablet	Neurology
**9**	Nonsteroidal Anti-inflammatory Drugs (NSAID)	Diclofenac	50 mg	Tablet	Pain and inflammation
**10**	Antidiabetic (Biguanide)	Metformin	500 mg	Tablet	Diabetes
**11**	Gastrointestinal Drug (Proton Pump Inhibitor)	Omeprazole	20 mg	Capsule	Digestive
**12**	Analgesic, Antipyretic	Acetaminophen	24 mg/ml	Suspension	Pain and inflammation
**13**	Respiratory Drug (Beta-2 Agonist)	Salbutamol	75 mg	Inhaler	Asthma and bronchitis
**14**	Lipid-lowering agent (selective cholesterol absorption inhibitors)	Ezetimibe	10 mg	Tablet	Cardiovascular

Appendix 1 14 WHO Core List of 14 Essential Medicines

Appendix 2 details all medicines analyzed in the study (14 WHO core + 37 Iran-specific additions), including Anatomical Therapeutic Chemical (ATC) codes, Defined Daily Doses (DDD), dosage form/DDD ratios, prescription quantities, disease types (acute/chronic), and estimated monthly usage. The data enables granular analysis of medication access patterns aligned with Iran's disease burden and treatment protocols.

Number	Generic code	Generic name	ATC code	DDD	Dose form/DDD	Number Per Prescription	Disease Type	Monthly Usage
**1**	68	Amitriptyline25mg	N06AA09	75mg	0.33	30	Chronic	90
**2**	77	Amoxicilline500mg	J01CA04	1.5gr	0.33	20	Acute	30
**3**	7125	Bisoprolol5mg	C07AB07	10mg	0.5	30	Chronic	60
**4**	205	Captopril25mg	C09AA01	50mg	0.5	90	Chronic	60
**5**	237	Ceftriaxone1gr	J01DD04	2gr	0.5	2	Acute	3
**6**	296	Ciprifloxacin500mg	J01MA02	1gr	0.5	20	Acute	20
**7**	340	Syrup Co-trimoxazol	J01EE01		1	1	Acute	1
**8**	420	Diazepam5mg	N05BA01	10mg	0.5	30	Acute	10
**9**	15917	Diclofenac50mg	M01AB05	0.1gr	0.5	20	Acute	20
**10**	817	Metformin500mg	A10BA02	2gr	0.25	100	Chronic	120
**11**	931	Omeprazole20mg	A02BC01	20mg	1	28	Acute	28
**12**	4	Syrup Acetaminophen60mg/ml	N02BE01	3gr	0.48	1	Acute	1

**13**	15068	Spray Salbutamol	R03AC02	0.8mg	25	1	Chronic	1
**14**	5657	Tab Ezetimibe	C10AX09	10mg	1	30	Chronic	30
**15**	2475	Carvedilol 6.25mg	C07AG02	37.5mg	0.16	90	Chronic	180
**16**	436	Digoxin.025mg	C01AA05	0.25mg	1	90	Chronic	30
**17**	2043	Enoxaparin4000	B01AB05	2tu	2	14	Acute	14
**18**	1933	Lozartan50mg	C09CA01	50mg	1	90	Chronic	30
**19**	1788	Clopidogrel75mg	B01AC04	75mg	1	90	Chronic	30
**20**	571	Furosemide40mg	C03CA01	40mg	1	90	Chronic	30
**21**	2099	Spironolactone100mg	C03DA01	75mg	1.33	90	Chronic	30
**22**	1395	Aspirin80mg	B01AC06	1tablet	1	90	Chronic	30
**23**	70	Amelodipine5mg	C08CA01	5mg	1	90	Chronic	30
**24**	7066	Propranolo20mg	C07AA05	0.16gr	0.12	60	Chronic	90
**25**	717	Isosorbide dinitrate10mg	C01DA08	60mg	0.16	90	Chronic	60
**26**	1910	Atorvastatin10mg	C10AA05	20mg	0.5	90	Chronic	60
**27**	50266	Hydrochloorthiazide12.5mg	C03AA03	25mg	0.5	90	Chronic	60
**28**	1328	Methylphenidate10mg	N06BA04	30mg	0.33	100	Chronic	90
**29**	52301	Levodopa - C 100/25mg	N04BA02	0.6gr	0.20	90	Chronic	144
**30**	334	Clozapine100mg	N05AH02	0.3gr	0.33	90	Chronic	90

**31**	767	Lithium carbonate300mg	N05AN01	24mmol	0.16	90	Chronic	177
**32**	277	Amp Chlorpromazine25mg/ml	N05AA01	0.1gr	0.25	1	Acute	4
**33**	1871	Sodium Valproate500mg	N03AG01	1.5gr	0.33	90	Chronic	90
**34**	40	Alprazolam 0.5mg	N05BA12	1mg	0.5	90	Chronic	60
**35**	1512	Fluoxetine10mg	N06AB03	20mg	0.5	30	Chronic	60
**36**	979	Phenobarbital100mg	N03AA02	0.1gr	1	90	Chronic	30
**37**	164	Biperiden2mg	N04AA02	10mg	0.2	90	Chronic	150
**38**	208	Carbamazepine200mg	N03AF01	1gr	0.2	90	Chronic	150
**39**	2465	Tamoxifen20mg	L02BA01	20mg	1	20	Chronic	30
**40**	751	Levothyroxine0.1mg	H03AA01	0.15mg	0.66	90	Chronic	45
**41**	7338	Insulin glargine	A10AE04	40u	7.5	3	Chronic	4
**42**	674	Insulin regular	A10AB01	40u	25	3	Chronic	1.2
**43**	1523	Gliclazide80mg	A10BB09	60mg	1.33	90	Chronic	22
**44**	595	Glibenclamide5mg	A10BB01	10mg	0.5	90	Chronic	60
**45**	1045	Prednisolone5mg	A01AC04	No DDDs have been established in this group	0.5	5	Chronic	60
**46**	400	Amp Dexamethasone8mg/ml	A01AC02	No DDDs have been establishe	5.33	1	Acute	4

				d in this group				
**47**	1182	Sulfasalazine500mg	A07EC01	2gr	0.25	90	Chronic	120
**48**	17336	Beclomethasone inhaler spray		0.8MG	25	1	Chronic	1.2
**49**	1424	Cetirizine10mg	R06AE07	10MG	1	20	Acute	20
**50**	1226	Timolol eye drop	S01ED01	-	25	1	Chronic	1.2
**51**	659	Ibuprofen400mg	M01AE01	1.2mg	0.33	20	Acute	20

Appendix 2 Comprehensive List of 51 Studied Medicines with Therapeutic and Utilization Metrics. Abbreviation: DDD: Defined Daily Doses

Appendix 3 reports the monthly Out of Pocket (OOP) expenditures for all 51 medicines, converted to USD using Purchasing Power Parity (PPP) exchange rates. Costs reflect medication procurement expenses (excluding administration fees) and highlight affordability challenges, with high-cost agents (e.g., enoxaparin: $152/month) contrasted against low-cost generics (e.g., diazepam: $0.41/month).

**Generic name**	**ATC code**	**2016 monthly cost**	**2017 monthly cost**	**2018 monthly cost**	**2019 monthly cost**	**2020 monthly cost**	**2021 monthly cost**	**2022 monthly cost**	**Average of the monthly cost**
**Amitriptyline25mg**	N06AA09	3.746	3.452	6.924	4.976	5.058	3.651	3.780	4.512
**Amoxicilline500mg**	J01CA04	6.602	6.241	3.980	2.893	3.793	3.199	5.912	4.660
**Bisoprolol5mg**	C07AB07	8.768	9.757	9.803	7.560	5.910	4.353	12.993	8.449
**Captopril25mg**	C09AA01	1.973	2.017	3.545	2.548	3.277	2.288	2.174	2.546
**Ceftriaxone1gr**	J01DD04	6.152	6.645	5.448	5.730	4.295	3.799	5.343	5.345
**Ciprifloxacin500mg**	J01MA02	3.599	3.523	7.783	6.202	3.699	2.525	3.533	4.409
**Syrup Co-trimoxazole**	J01EE01	2.281	2.391	1.986	1.427	1.647	1.089	2.758	1.940

**Diazepam5mg**	N05BA01	0.398	0.371	0.650	0.467	0.466	0.311	0.254	0.417
**Diclofenac50mg**	M01AB05	0.953	1.404	1.539	1.444	1.062	0.723	0.532	1.094
**Metformin500mg**	A10BA02	9.155	8.728	8.594	8.704	9.304	6.562	11.475	8.932
**Omeprazole20mg**	A02BC01	3.957	4.032	4.418	3.688	3.008	2.241	2.447	3.399
**Syrup Acetaminophen60mg/ml**	N02BE01	1.784	2.411	2.774	2.209	2.164	1.738	2.301	2.197
**Spray Salbutamol**	R03AC02	9.073	9.186	7.230	6.281	7.147	5.889	6.121	7.275
**Tab Ezetimibe**	C10AX09	5.111	5.517	4.964	3.822	3.004	2.236	3.025	3.954
**Carvedilol 6.25mg**	C07AG02	10.986	11.618	11.966	10.541	8.455	7.975	7.927	9.924
**Digoxin.025mg**	C01AA05	1.795	1.945	1.787	1.609	1.639	1.105	1.491	1.625
**Enoxaparin4000**	B01AB05	162.348	156.283	136.146	144.727	122.100	163.710	179.678	152.142
**Lozartan50mg**	C09CA01	2.586	2.981	2.666	2.252	2.264	1.931	1.771	2.350
**Clopidogrel75mg**	B01AC04	2.477	2.573	7.564	5.733	8.593	5.757	20.922	7.660
**Furosemide40mg**	C03CA01	1.816	1.867	1.581	1.260	1.193	0.870	0.903	1.356
**Spironolactone100mg**	C03DA01	1.068	2.370	6.267	4.561	5.644	3.947	8.238	4.585
**Aspirin80mg**	B01AC06	1.376	2.090	2.468	2.032	1.748	1.275	1.111	1.728
**Amelodipine5mg**	C08CA01	1.923	2.049	2.221	1.741	1.508	1.109	1.077	1.661
**Propranolo20mg**	C07AA05	2.762	3.239	6.403	4.601	4.514	3.066	2.563	3.878
**Isosorbide dinitrate10mg**	C01DA08	1.999	2.311	2.361	1.947	1.753	1.910	2.133	2.059
**Atorvastatin10mg**	C10AA05	4.471	4.837	4.194	3.506	3.139	2.746	2.451	3.621
**Hydrochloorthiazide12.5mg**	C03AA03	0.931	1.181	1.790	1.483	1.551	1.098	0.963	1.285
**Methylphenidate10mg**	N06BA04	18.561	19.480	15.710	11.553	8.535	6.305	7.006	12.450
**Levodopa -C 100/25mg**	N04BA02	15.197	17.740	17.656	15.141	15.731	37.372	31.169	21.429
**Clozapine100mg**	N05AH02	20.408	20.424	17.263	14.339	12.598	8.476	11.626	15.019
**Lithium carbonate300mg**	N05AN01	20.105	27.076	57.525	43.579	42.384	28.397	30.123	35.599

**Amp Chlorpromazine25 mg/ml**	N05AA01	4.754	6.559	5.560	4.475	3.766	3.290	3.036	4.492
**Sodium Valproate500mg**	N03AG01	32.271	31.863	32.744	25.178	22.247	14.796	31.764	27.266
**Alprazolam 0.5mg**	N05BA12	3.209	3.064	3.460	2.586	2.538	1.711	2.005	2.653
**Fluoxetine10mg**	N06AB03	3.418	3.262	3.439	2.477	3.275	4.201	4.376	3.492
**Phenobarbital100mg**	N03AA02	1.592	1.766	1.750	1.357	1.645	1.141	2.384	1.662
**Biperiden2mg**	N04AA02	13.961	13.965	13.508	10.198	13.282	9.210	11.486	12.230
**Carbamazepine200 mg**	N03AF01	15.703	16.802	15.299	12.287	14.834	13.999	17.070	15.142
**Tamoxifen20mg**	L02BA01	3.759	4.674	6.208	5.886	4.573	4.410	2.507	4.574
**Levothyroxine0.1mg**	H03AA01	3.761	3.797	3.462	3.107	2.991	2.309	2.152	3.083
**Insulin glargine**	A10AE04	99.542	90.348	71.203	53.092	39.397	34.270	39.114	60.995
**Insulin regular**	A10AB01	12.615	11.253	10.405	8.221	7.854	5.371	4.810	8.647
**Gliclazide80mg**	A10BB09	1.102	1.490	1.673	1.330	1.333	0.947	2.378	1.465
**Glibenclamide5mg**	A10BB01	2.080	3.500	3.545	2.869	2.485	1.715	1.395	2.513
**Prednisolone5mg**	A01AC04	3.626	3.358	4.492	3.725	3.421	2.403	2.373	3.343
**Amp Dexamethasone8mg /ml**	A01AC02	3.243	3.055	3.808	2.980	2.617	2.034	2.020	2.822
**Sulfasalazine500mg**	A07EC01	12.664	15.472	13.948	10.538	15.192	11.359	14.141	13.331
**Beclomethasone inhaler spray**		1.263	1.213	1.002	8.273	8.622	5.970	5.232	4.511
**Cetirizine10mg**	R06AE07	1.260	1.770	1.539	1.211	1.138	0.801	0.883	1.229
**Timolol eye drop**	S01ED01	2.722	2.605	2.145	1.638	1.529	1.579	1.293	1.930
**Ibuprofen400mg**	M01AE01	1.466	1.527	1.340	1.145	2.451	3.384	2.658	1.996

Appendix 3 Calculated Monthly Out-of-Pocket (OOP) Costs for Essential Medicines in Iran (2016–2022, PPP-adjusted USD)

Appendix 4 summarizes Average Annual Percentage Change (AAPC) in OOP payments under Iran's two primary insurers. Statistically significant trends ($p<0.05$) are flagged, revealing rising burdens

for drugs like ezetimibe and declining costs for insulin glargine. Confidence intervals and significance markers clarify policy-relevant variations in financial accessibility.

Drug	Insurance (0=SSO, 1=IHIO)	Joinpoint Model	Start	End	AAPC	AAPC C.I. Low	AAPC C.I. High	Statistically Significant (0=No, 1=Yes)
**Amitriptyline25mg**	0	0	2016	2022	-1.5124	-4.0697	1.1168	0
**Amoxicilline500mg**	0	1	2016	2022	-7.5389	-13.2829	0.0189	0
**Bisoprolol5mg**	0	0	2016	2022	-2.9101	-9.3259	4.1029	0
**Captopril25mg**	0	0	2016	2022	-0.8313	-4.1011	2.7059	0
**Ceftriaxone1gr**	0	0	2016	2022	0.8955	-2.7644	4.7541	0
**Ciprifloxacin500mg**	0	0	2016	2022	-2.9272	-10.7861	5.9152	0
**Syrup Co-trimoxazole**	0	0	2016	2022	-1.3597	-6.2674	3.9635	0
**Diazepam5mg**	0	0	2016	2022	-0.8751	-7.3493	6.1839	0
**Diclofenac50mg**	0	1	2016	2022	1.1146	-0.4256	2.6978	0
**Metformin500mg**	0	1	2016	2022	-8.8065	13.9405	-1.7287	1
**Omeprazole20mg**	0	0	2016	2022	-2.9305	-7.3618	1.7861	0
**Syrup Acetaminophen60 mg/ml**	0	0	2016	2022	-4.8493	-9.5452	0.1602	0
**Spray Salbutamol**	0	0	2016	2022	-1.8341	-3.6642	0.0075	0
**Tab Ezetimibe**	0	0	2016	2022	2.704	0.3162	5.1161	1
**Carvedilol 6.25mg**	0	1	2016	2022	-3.3647	-6.6817	0.3433	0
**Digoxin.025mg**	0	1	2016	2022	7.5421	2.5119	11.4336	1
**Enoxaparin4000**	0	1	2016	2022	-4.0911	-5.6	-2.5572	1
**Lozartan50mg**	0	1	2016	2022	-4.7983	-9.1269	0.5109	0
**Clopidogrel75mg**	0	1	2016	2022	-6.3706	-9.8263	-1.3633	1
**Furosemide40mg**	0	0	2016	2022	0.5622	-4.1944	5.6362	0
**Spironolactone100 mg**	0	1	2016	2022	8.2914	-0.6978	15.7987	0
**Aspirin80mg**	0	0	2016	2022	-4.1522	-11.515	4.0364	0
**Amelodipine5mg**	0	1	2016	2022	-1.2346	-5.3022	3.7409	0
**Propranolo20mg**	0	0	2016	2022	-2.7243	-4.5024	-0.9423	1

**Isosorbide dinitrate10mg**	0	1	2016	2022	-2.7737	-5.2191	-0.4552	1
**Atorvastatin10mg**	0	1	2016	2022	-1.1678	-6.4833	3.8082	0
**Hydrochloorthiazide12.5mg**	0	0	2016	2022	-4.0466	12.6113	5.4115	0
**Methylphenidate10mg**	0	0	2016	2022	-3.6065	-8.8921	2.0526	0
**Levodopa - C 100/25mg**	0	0	2016	2022	-0.0836	-2.9214	2.7865	0
**Clozapine100mg**	0	0	2016	2022	-3.4829	-8.4248	1.8071	0
**Lithium carbonate300mg**	0	0	2016	2022	-7.6059	12.9223	-1.8346	1
**Amp Chlorpromazine25 mg/ml**	0	0	2016	2022	1.4468	0.7409	2.1796	1
**Sodium Valproate500mg**	0	0	2016	2022	-5.3084	-9.6033	-0.6918	1
**Alprazolam 0.5mg**	0	0	2016	2022	-0.9193	-5.4775	3.9576	0
**Fluoxetine10mg**	0	1	2016	2022	-6.1918	-9.1743	-2.7695	1
**Phenobarbital100 mg**	0	0	2016	2022	-5.3598	-14.2288	4.8902	0
**Biperiden2mg**	0	1	2016	2022	-6.6874	-11.0277	-1.4489	1
**Carbamazepine200mg**	0	1	2016	2022	-6.9693	-12.3597	-0.1241	1
**Tamoxifen20mg**	0	1	2016	2022	0.0928	-0.4552	0.6097	0
**Levothyroxine0.1 mg**	0	1	2016	2022	-4.7297	-9.8816	1.6161	0
**Insulin glargine**	0	1	2016	2022	-3.1987	-5.2662	-0.4412	1
**Insulin regular**	0	1	2016	2022	-3.2203	-5.5044	-0.1694	1
**Gliclazide80mg**	0	0	2016	2022	-3.7874	-6.7224	-0.7177	1
**Glibenclamide5mg**	0	0	2016	2022	-2.1345	-7.6075	3.6433	0
**Prednisolone5mg**	0	0	2016	2022	0.0003	-2.6806	2.8016	0
**Amp Dexamethasone8mg/ml**	0	0	2016	2022	0.4116	-1.2214	2.0901	0
**Sulfasalazine500mg**	0	1	2016	2022	-2.7713	-4.9184	-0.6292	1
**Beclomethasone inhaler spray**	0	0	2016	2022	-10.9633	-18.936	-2.3077	1
**Cetirizine10mg**	0	0	2016	2022	-1.379	-4.6424	2.0409	0
**Timolol eye drop**	0	1	2016	2022	0.4276	-0.8277	1.5793	0

**Ibuprofen400mg**	0	1	2016	2022	-6.5962	-10.0335	-2.6511	1
**Amitriptyline25mg**	1	0	2016	2022	-1.5124	-4.0697	1.1168	0
**Amoxicilline500mg**	1	1	2016	2022	-7.9734	-13.7556	-0.1734	1
**Bisoprolol5mg**	1	0	2016	2022	-2.9101	-9.3259	4.1029	0
**Captopril25mg**	1	0	2016	2022	-0.8313	-4.1011	2.7059	0
**Ceftriaxone1gr**	1	0	2016	2022	0.8955	-2.7644	4.7541	0
**Ciprifloxacin500mg**	1	0	2016	2022	-2.9272	-10.7861	5.9152	0
**Syrup Co-trimoxazole**	1	1	2016	2022	-8.2155	-13.2325	-1.1636	1
**Diazepam5mg**	1	0	2016	2022	-0.8751	-7.3493	6.1839	0
**Diclofenac50mg**	1	0	2016	2022	0.2358	-3.6991	4.3523	0
**Metformin500mg**	1	1	2016	2022	-8.8065	-13.9405	-1.7287	1
**Omeprazole20mg**	1	0	2016	2022	-2.9305	-7.3618	1.7861	0
**Syrup Acetaminophen60 mg/ml**	1	0	2016	2022	-4.8493	-9.5452	0.1602	0
**Spray Salbutamol**	1	0	2016	2022	-1.8341	-3.6642	0.0075	0
**Tab Ezetimibe**	1	0	2016	2022	2.704	0.3162	5.1161	1
**Carvedilol 6.25mg**	1	1	2016	2022	-3.3647	-6.6817	0.3433	0
**Digoxin.025mg**	1	0	2016	2022	-3.7722	-11.108	4.1995	0
**Enoxaparin4000**	1	1	2016	2022	-4.0911	-5.6	-2.5572	1
**Lozartan50mg**	1	1	2016	2022	-3.7672	-7.1075	0.2047	0
**Clopidogrel75mg**	1	1	2016	2022	-6.3706	-9.8263	-1.3633	1
**Furosemide40mg**	1	0	2016	2022	0.5622	-4.1944	5.6362	0
**Spironolactone100 mg**	1	0	2016	2022	-6.5417	-10.0712	-2.7023	1
**Aspirin80mg**	1	0	2016	2022	-4.1522	-11.515	4.0364	0
**Amelodipine5mg**	1	1	2016	2022	-1.2346	-5.3022	3.7409	0
**Propranolo20mg**	1	0	2016	2022	-2.7243	-4.5024	-0.9423	1
**Isosorbide dinitrate10mg**	1	1	2016	2022	-2.7737	-5.2191	-0.4552	1
**Atorvastatin10mg**	1	1	2016	2022	-1.1678	-6.4833	3.8082	0
**Hydrochloorthiazide12.5mg**	1	0	2016	2022	-4.0466	-12.6113	5.4115	0
**Methylphenidate10mg**	1	0	2016	2022	-3.6065	-8.8921	2.0526	0

**Levodopa - C 100/25mg**	1	0	2016	2022	-0.0836	-2.9214	2.7865	0
**Clozapine100mg**	1	0	2016	2022	-3.4829	-8.4248	1.8071	0
**Lithium carbonate300mg**	1	0	2016	2022	-7.6059	-12.9223	-1.8346	1
**Amp Chlorpromazine25 mg/ml**	1	0	2016	2022	1.4468	0.7409	2.1796	1
**Sodium Valproate500mg**	1	0	2016	2022	-5.3084	-9.6033	-0.6918	1
**Alprazolam 0.5mg**	1	0	2016	2022	-0.9193	-5.4775	3.9576	0
**Fluoxetine10mg**	1	1	2016	2022	-6.1918	-9.1743	-2.7695	1
**Phenobarbital100 mg**	1	0	2016	2022	-5.3598	-14.2288	4.8902	0
**Biperiden2mg**	1	1	2016	2022	-6.6874	-11.0277	-1.4489	1
**Carbamazepine200mg**	1	1	2016	2022	-6.9693	-12.3597	-0.1241	1
**Tamoxifen20mg**	1	0	2016	2022	-3.6661	-6.3152	-0.9089	1
**Levothyroxine0.1 mg**	1	1	2016	2022	-4.7297	-9.8816	1.6161	0
**Insulin glargine**	1	1	2016	2022	-12.5993	-19.3612	-3.2861	1
**Insulin regular**	1	1	2016	2022	-12.3934	-18.9345	-3.2201	1
**Gliclazide80mg**	1	1	2016	2022	7.5143	0.4606	13.2665	1
**Glibenclamide5mg**	1	0	2016	2022	-2.1345	-7.6075	3.6433	0
**Prednisolone5mg**	1	0	2016	2022	0.0003	-2.6806	2.8016	0
**Amp Dexamethasone8mg/ml**	1	0	2016	2022	0.4116	-1.2214	2.0901	0
**Sulfasalazine500mg**	1	1	2016	2022	-2.7713	-4.9184	-0.6292	1
**Beclomethasone inhaler spray**	1	0	2016	2022	-10.9633	-18.936	-2.3077	1
**Cetirizine10mg**	1	0	2016	2022	-1.3917	-4.6772	2.0557	0
**Timolol eye drop**	1	1	2016	2022	0.4276	-0.8277	1.5793	0
**Ibuprofen400mg**	1	1	2016	2022	-6.5962	-10.0335	-2.6511	1

Appendix 4 Joinpoint Regression Analysis of Annual OOP Payment Trends by Medicine and Insurance Type (2016–2022) Abbreviation: SSO: Social Security Organization, IHIO: Iran Health Insurance Organization.

Generic name	2016		2017	
	SSO	IHIO	SSO	IHIO
Acetaminophen 60mg	50.50%	43.10%	52.60%	47.30%
Alperazolam 0.5mg	47.20%	42.50%	46.70%	53.40%
Amelodipine5mg	44.30%	39.40%	41.50%	36.10%
Amitriptyline5mg2	58.80%	57.20%	64.30%	71.60%
Amoxicilline500mg	42.90%	37.30%	45.60%	37.70%
Amp Chlorpromazine	74.80%	43.00%	72.10%	45.20%
Aspirin80mg	49.60%	48.20%	43.50%	45.80%
Atorvastatin10mg	42.50%	58.20%	53.40%	59.60%
Beclomethasone inhal	62.80%	42.10%	66.20%	48.20%
Biperiden2mg	48.30%	57.20%	45.30%	55.80%
Bisoprolol5mg	43.00%	74.80%	45.20%	72.10%
Captopril25mg	45.20%	54.80%	50.30%	69.90%
Carbamazepine200mg	37.60%	62.80%	44.00%	66.20%
Carvedilol 6.25mg	39.90%	51.70%	46.50%	48.00%
Ceftriaxone1gr	67.00%	45.20%	67.10%	50.30%
Cetirizine10mg	60.30%	51.90%	62.40%	54.20%
Ciprifloxacin500mg	42.10%	48.90%	48.20%	45.60%
Clopidogrel75mg	57.20%	50.50%	42.40%	52.60%
Clozapine100mg	38.70%	81.60%	37.80%	83.70%
Co-trimoxazol (Syrup	51.90%	38.50%	54.20%	47.80%
Dexamethasone 8mg/	81.60%	58.80%	83.70%	64.30%
Diazepam5mg	57.20%	41.50%	71.60%	45.20%
Diclofenac50mg	63.40%	67.00%	60.60%	67.10%
Digoxin.025mg	40.50%	38.90%	45.20%	38.60%
Enoxaparin4000	41.10%	53.60%	44.20%	50.50%
Ezetimibe (Tab)	41.10%	43.40%	43.50%	42.00%
Fluoxetine10mg	58.20%	41.40%	59.60%	40.70%
Furosemide40mg	43.10%	42.50%	47.30%	42.80%
Glibenclamide5mg	57.20%	41.10%	48.60%	43.50%
Gliclazide80mg	51.70%	57.20%	48.00%	42.40%
Hydrochloorthiazide1	54.80%	39.90%	69.90%	46.50%
Ibuprofen400mg	58.40%	63.40%	59.90%	60.60%
Insulin glargine	36.30%	52.40%	37.20%	53.90%
Insulin regular	38.90%	79.70%	38.60%	85.00%
Isosorbide dinitrate10	49.00%	49.60%	50.90%	43.50%
Levodopa - C 100/25	34.80%	58.40%	39.60%	59.90%
Levothyroxine0.1mg	43.40%	38.70%	42.00%	37.80%
Lithium carbonate300	42.50%	47.20%	42.80%	46.70%
Lozartan50mg	38.50%	41.10%	47.80%	42.00%
Metformin500mg	41.40%	37.60%	40.70%	44.00%
Methylphenidate10mg	39.40%	57.20%	36.10%	48.60%
Omeprazole20mg	44.20%	34.80%	50.20%	39.60%
Phenobarbital100mg	48.90%	44.30%	45.60%	41.50%
Prednisolone5mg	79.70%	42.90%	85.00%	45.60%
Propranolo20mg	57.20%	44.20%	55.80%	50.20%

Salbutamol (Spray)	41.50%	48.30%	45.20%	45.30%
Sodium Valproate500	37.30%	60.30%	37.70%	62.40%
Spironolactone100mg	48.20%	49.00%	45.80%	50.90%
Sulfasalazine500mg	41.10%	36.30%	42.00%	37.20%
Tamoxifen20mg	53.60%	40.50%	50.50%	45.20%
Timolol eye drop	52.40%	41.10%	53.90%	44.20%

Out-of-Pocket (OOP) Percentages for Srudied Medicines (2016-2022). Abbreviation: SSO: Social Security Organi

2018		2019		202[illegible]
**SSO**	**IHIO**	**SSO**	**IHIO**	**SSO**
50.30%	47.80%	50.20%	46.40%	45.00%
44.80%	51.50%	46.60%	47.40%	49.60%
45.30%	38.50%	46.80%	37.30%	48.40%
56.40%	59.90%	55.00%	59.80%	52.90%
51.40%	34.60%	54.40%	35.40%	46.10%
75.70%	45.80%	77.10%	46.00%	77.20%
43.90%	35.80%	51.60%	39.70%	42.00%
51.50%	57.70%	47.40%	59.50%	43.50%
71.70%	40.10%	44.20%	39.10%	40.30%
42.20%	50.50%	45.00%	52.00%	40.40%
45.80%	75.70%	46.00%	77.10%	43.90%
45.80%	56.80%	47.10%	52.60%	42.00%
40.40%	71.70%	42.20%	44.20%	37.20%
49.10%	47.70%	48.60%	49.30%	50.20%
70.90%	45.80%	68.70%	47.10%	72.00%
61.90%	54.80%	63.10%	56.60%	58.80%
40.10%	44.10%	39.10%	49.10%	41.20%
36.00%	50.30%	35.50%	50.20%	33.60%
35.80%	81.00%	37.20%	83.40%	36.40%
54.80%	41.20%	56.60%	45.30%	49.80%
81.00%	56.40%	83.40%	55.00%	83.10%
59.90%	43.20%	59.80%	41.80%	54.40%
60.30%	70.90%	57.80%	68.70%	58.20%
46.50%	40.30%	44.00%	41.80%	42.00%
42.70%	47.20%	43.60%	44.20%	39.80%
43.70%	43.20%	44.00%	45.10%	45.40%
57.70%	41.50%	59.50%	40.40%	55.00%
47.80%	33.50%	46.40%	35.20%	49.90%
46.80%	43.70%	49.40%	44.00%	49.50%
47.70%	36.00%	49.30%	35.50%	42.80%
56.80%	49.10%	52.60%	48.60%	46.00%
65.80%	60.30%	65.40%	57.80%	74.50%
37.60%	57.10%	39.70%	56.70%	37.40%
40.30%	82.10%	41.80%	82.30%	39.40%
59.60%	43.90%	54.50%	51.60%	48.80%
39.10%	65.80%	39.10%	65.40%	38.10%
43.20%	35.80%	45.10%	37.20%	42.50%
33.50%	44.80%	35.20%	46.60%	34.80%
41.20%	42.00%	45.30%	43.00%	44.00%
41.50%	40.40%	40.40%	42.20%	39.40%
38.50%	46.80%	37.30%	49.40%	38.00%
46.30%	39.10%	45.20%	39.10%	43.60%
44.10%	45.30%	49.10%	46.80%	43.90%
82.10%	51.40%	82.30%	54.40%	78.30%
50.50%	46.30%	52.00%	45.20%	52.10%

43.20%	42.20%	41.80%	45.00%	41.70%
34.60%	61.90%	35.40%	63.10%	33.20%
35.80%	59.60%	39.70%	54.50%	35.00%
42.00%	37.60%	43.00%	39.70%	39.30%
47.20%	46.50%	44.20%	44.00%	42.30%
57.10%	42.70%	56.70%	43.60%	52.40%

ization, IHIO: Iran Health Insurance Organization.

20	2021		2022	
IHIO	SSO	IHIO	SSO	IHIO
49.90%	51.30%	57.50%	33.50%	39.30%
43.50%	55.30%	46.60%	37.40%	41.80%
38.00%	49.00%	41.60%	37.10%	25.60%
54.40%	59.60%	64.50%	54.90%	58.30%
33.20%	50.80%	35.40%	23.90%	23.70%
43.90%	79.40%	52.60%	79.60%	29.90%
35.00%	45.70%	36.50%	32.80%	30.00%
55.00%	46.60%	52.30%	41.80%	38.50%
41.20%	41.30%	47.20%	35.20%	32.00%
52.10%	40.50%	53.20%	29.30%	45.20%
77.20%	52.60%	79.40%	29.90%	79.60%
46.00%	48.10%	54.50%	44.40%	47.20%
40.30%	37.20%	41.30%	24.40%	35.20%
42.80%	44.70%	45.30%	33.30%	91.80%
42.00%	73.50%	48.10%	68.10%	44.40%
49.80%	65.70%	58.60%	52.10%	26.60%
43.90%	47.20%	47.80%	32.00%	28.40%
45.00%	35.00%	51.30%	40.70%	33.50%
83.10%	38.60%	86.50%	27.20%	82.20%
44.00%	58.60%	39.50%	44.70%	32.50%
52.90%	86.50%	59.60%	82.20%	54.90%
41.70%	64.50%	41.80%	58.30%	37.20%
72.00%	68.20%	73.50%	65.70%	68.10%
39.40%	51.20%	41.40%	68.90%	13.90%
42.30%	38.10%	46.40%	31.50%	41.50%
42.50%	53.70%	46.70%	45.20%	28.50%
39.40%	52.30%	43.60%	38.50%	19.80%
34.80%	57.50%	35.20%	39.30%	22.90%
45.40%	52.40%	53.70%	43.60%	45.20%
33.60%	45.30%	35.00%	38.90%	40.70%
50.20%	54.50%	44.70%	47.20%	33.30%
58.20%	44.80%	68.20%	41.70%	60.60%
52.40%	38.70%	54.20%	28.20%	54.70%
78.30%	41.40%	85.60%	29.70%	80.60%
42.00%	48.30%	45.70%	38.10%	32.80%
74.50%	41.40%	44.80%	33.90%	41.70%
36.40%	46.70%	38.60%	28.50%	27.20%
49.60%	35.20%	55.30%	22.90%	37.40%
39.30%	39.50%	41.40%	29.90%	32.60%
37.20%	43.60%	37.20%	19.80%	24.40%
49.50%	41.60%	52.40%	25.60%	43.60%
38.10%	48.70%	41.40%	34.80%	33.90%
48.40%	47.80%	49.00%	28.40%	37.10%
46.10%	85.60%	50.80%	80.60%	23.10%
43.60%	53.20%	48.70%	45.20%	34.80%

40.40%	41.80%	40.50%	37.20%	29.30%
58.80%	35.40%	65.70%	23.70%	52.00%
48.80%	36.50%	48.30%	92.30%	38.10%
37.40%	41.40%	38.70%	32.60%	12.90%
42.00%	46.40%	51.20%	54.50%	26.90%
39.80%	54.20%	38.10%	54.70%	31.50%
